# Deep Neural Network Modeling of Circular RNA‐Mediated Epitranscriptomic Modifications in Alzheimer's Disease Progression: A Multi‐Omic Approach Using Single‐Nucleus RNA Sequencing and Spatial Epigenomics

**DOI:** 10.1002/alz70855_102535

**Published:** 2025-12-23

**Authors:** Rifaldy Fajar, Prihantini Prihantini, Sahnaz V Putri

**Affiliations:** ^1^ Yogyakarta State University, Sleman, Special Region of Yogyakarta, Indonesia; ^2^ The Integrated Mathematical, Computational, and Data Science for BioMedicine Research Community Foundation, Jakarta Barat, DKI Jakarta, Indonesia; ^3^ Bandung Institute of Technology, Bandung, West Java, Indonesia; ^4^ International University Semen Indonesia, Gresik, East Java, Indonesia

## Abstract

**Background:**

Circular RNAs (circRNAs) are increasingly recognized as significant regulators of gene expression and RNA‐protein interactions, with growing evidence indicating that their epitranscriptomic modifications play a role in neurodegenerative processes. However, their involvement in Alzheimer's disease (AD) remains insufficiently understood. This study utilizes single‐nucleus RNA sequencing (snRNA‐seq) and spatial epigenomics to explore circRNA‐mediated regulatory mechanisms in AD. By applying a deep neural network (DNN) framework, the research aims to identify circRNA‐driven molecular changes and their effects on neuronal and glial function, providing valuable advancements in understanding AD pathogenesis.

**Method:**

Data were obtained from the Allen Institute for Brain Science's Aging, Dementia, and TBI snRNA‐seq dataset, which includes 107 brain samples and 377 tissue sections from cortical gray matter, white matter, and hippocampal regions. Epigenomic profiles were sourced from the NIH Roadmap Epigenomics Project for histone modifications and chromatin accessibility in brain tissues. High‐confidence circRNA isoforms were annotated using CIRIquant and mapped to RNA modifications (m6A, m5C) derived from spatial epigenomics. A customized DNN model, CircEpiNet, was developed to predict phenotypic impacts of circRNA modifications on synaptic, mitochondrial, and inflammatory pathways. Multi‐omic integration localized circRNA activity, and findings were validated using snRNA‐seq data from the AMP‐AD Knowledge Portal (ROSMAP dataset).

**Result:**

The CircEpiNet model identified 14 circRNAs with significant epitranscriptomic modifications in AD. For example, circAPP hyper‐methylation (m6A) disrupted amyloid precursor protein processing (effect size = 0.71, 95% CI: 0.68–0.74), and circTREM2 hyper‐methylation (m5C) correlated with microglial activation (*r* = 0.79, *p* < 0.001). circPGC1A hypo‐methylation reduced mitochondrial biogenesis in 65.4% of early‐stage AD nuclei (95% CI: 62.1–68.3%). The DNN model achieved an AUROC of 0.87 (95% CI: 0.84–0.90) in predicting cellular phenotypes.

**Conclusion:**

This study identifies the essential roles of circRNAs in Alzheimer's disease progression using a robust multi‐omic deep learning framework. By integrating snRNA‐seq and spatial epigenomics, the findings contribute to a deeper understanding of potential therapeutic targets for Alzheimer's disease.

